# Inhibitory effect of (pro)renin receptor decoy inhibitor PRO20 on endoplasmic reticulum stress during cardiac remodeling

**DOI:** 10.3389/fphar.2022.940365

**Published:** 2022-08-12

**Authors:** Jing Zhang, Yun-Jiu Cheng, Chang-Jun Luo, Jia Yu

**Affiliations:** ^1^ Department of Cardiology, Liuzhou Municipal Liutie Central Hospital, Liuzhou, China; ^2^ Department of Cardiology, The First Affiliated Hospital of Sun Yat-sen University, Guangzhou, China; ^3^ Department of General Practice School, Guangxi Medical University, Nanning, China

**Keywords:** (Pro)renin receptor, PRO20, heart failure, cardiac remodeling, endoplasmic reticulum stress

## Abstract

**Background:** Ectopic activation of renin-angiotensin-system contributes to cardiovascular and renal diseases. (Pro)renin receptor (PRR) binds to renin and prorenin, participating in the progression of nephrology. However, whether PRR could be considered as a therapeutic target for cardiac remodeling and heart failure remains unknown.

**Materials and methods:** Transverse aortic constriction (TAC) surgery was performed to establish a mouse model of chronic pressure overload-induced cardiac remodeling. Neonatal rat cardiomyocytes (CMs) and cardiac fibroblasts (CFs) were isolated and stimulated by Angiotensin II (Ang II). PRR decoy inhibitor PRO20 was synthesized and used to evaluate its effect on cardiac remodeling.

**Results:** Soluble PRR and PRR were significantly upregulated in TAC-induced cardiac remodeling and Ang II-treated CMs and CFs. Results of *In vivo* experiments showed that suppression of PRR by PRO20 significantly retarded cardiac remodeling and heart failure indicated by morphological and echocardiographic analyses. *In vitro* experiments, PRO20 inhibited CM hypertrophy, and also alleviated CF activation, proliferation and extracellular matrix synthesis. Mechanically, PRO20 enhanced intracellular cAMP levels, but not affected cGMP levels in CMs and CFs. Moreover, treatment of PRO20 in CFs markedly attenuated the production of reactive oxygen species and phosphorylation of IRE1 and PERK, two well-identified markers of endoplasmic reticulum (ER) stress. Accordingly, administration of PRO20 reversed ER stressor thapsigargin-induced CM hypertrophy and CF activation/migration.

**Conclusion:** Taken together, these findings suggest that inhibition of PRR by PRO20 attenuates cardiac remodeling through increasing cAMP levels and reducing ER stress in both CMs and CFs.

## Introduction

Cardiac remodeling is the common pathological change in the onset and development of multiple cardiovascular diseases, including hypertension, ischemic cardiomyopathy and non-ischemic cardiomyopathy (Abboud and Januzzi, 2021). During the pathological process, cardiac hypertrophy is characterized by increased cardiomyocyte (CM) size, enhanced protein synthesis and excessive organization of the sarcomere (Yang et al., 2020). Cardiac fibrosis, as a scarring event in the cardiac muscle, is characterized by net accumulation of extracellular matrix in the myocardium, mediated by cardiac fibroblast (CF) activation and differentiation into myofibroblasts (Yang et al., 2020; Frangogiannis, 2021). Thus, early inhibition and even reversal of cardiac remodeling is an effective therapeutic regime for retarding heart failure (HF).

Current knowledge proves that the renin-angiotensin system (RAS) plays a central role in the pathogenesis of cardiac remodeling and HF (Abboud and Januzzi, 2021). Inhibition of RAS by angiotensin-converting enzyme inhibitors (ACEIs) and angiotensin receptor blockers (ARBs) has been approved for the treatment of numerous cardiovascular diseases including hypertension, atherosclerosis and HF due to its beneficial effect on cardiovascular remodeling (Singh and Karnik, 2016). Prorenin receptor (PRR), a new component of the RAS, serves as a specific receptor for prorenin and renin to regulate their catalytic activity (Manerikar et al., 1976). Furthermore, the serum concentration of soluble PRR is a potential biomarker of tissue RAS activity (Fu et al., 2021). Previous studies provided full insights into the distribution and function of PRR in kidney diseases (Chen and Xu, 2020; Wang et al., 2020; Arthur et al., 2021). Accordingly, PRR decoy inhibitor PRO20 attenuated albumin overload-induced nephropathy in rats (Fang et al., 2018). However, the role of PRR decoy inhibitor in cardiac remodeling and HF remains unclear.

Endoplasmic reticulum (ER) is an organelle that functions in the folding, transport and secretion of lumen and membrane proteins and maintenance of intracellular calcium balance. Disruption of ER homeostasis provokes ER stress and causes the activation of downstream signaling such as inositol requiring enzyme 1 (IRE-1), PKR-like eukaryotic initiation factor 2a kinase (PERK), activating transcription factor-6 (ATF6) (Wang et al., 2018). Activation of unfolded protein response leads to ER stress via phosphorylation of IRE1 and PERK, or translocating ATF-6 to the Golgi apparatus and generating cleaved N-terminal cytoplasmic domain of ATF-6 (ATF-6 [N]) (Wang and Kaufman, 2016). Recent studies have indicated that ER stress is an essential signaling cascade for cardiovascular diseases (Wang et al., 2018; Zhang et al., 2020).

In the present study, we first assessed the plasma concentration of soluble PRR in mice with pressure overload and the expression of PRR in mouse failing hearts. Next, we investigated the effects of PRR blocker PRO20 on cardiac remodeling and cardiac dysfunction induced by transverse aortic contraction (TAC) in mice. In addition, we determined the underlying mechanism of PRO20 in CMs and CFs during cardiac remodeling. This study may extend our understanding of PRR and provide a new potential therapeutic target for HF.

## Materials and methods

### Mouse model

All experimental procedures involving animals were performed in accordance with the guidelines of the National Institutes of Health for the care and use of laboratory animals (NIH Publication, eighth Edition, 2011) and approved by the Animal Care and Use Committees of Liuzhou Municipal Liutie Central Hospital. TAC surgery was conducted to induce pathological cardiac hypertrophy *in vivo*. Briefly, 8-week-old C57BL/6J mice were anesthetized via intraperitoneal injections of a xylazine (5 mg/kg) and ketamine (80 mg/kg) mixture, and the aortic arch was visualized and ligated with 6–0 silk suture against a 27-gauge needle to form an approximately 70% aortic constriction. The needle was removed and the chest was closed with 5–0 silk suture (Xu et al., 2019). In order to improve the stability and reproducible of animal model, we arranged an experienced technician to perform TAC surgery. Three days prior to TAC surgery, PRO20 dissolved in saline was administered at 250 μg/kg/d or 500 μg/kg/d *via* subcutaneously implanted osmotic minipump according to previous reports (Wang et al., 2020). Equal volume of saline was administrated via subcutaneously implanted osmotic minipump in control group (Wang et al., 2019). At the end of the experiments, the mice were sacrificed by intraperitoneally injection of a lethal dose of pentobarbital sodium (100 mg/kg) to obtain the blood and heart for further analysis.

### Echocardiographic analysis

Using B-mode imaging (Vevo770; VisualSonics, Canada), the transducer (30-MHz RMV707B) was positioned to image longitudinal or cross sections of the heart. All echocardiographic measurements were made 3 times and the average values were reported. We finally analyzed the fractional shortening (FS), left ventricular ejection fraction (LVEF), left ventricular internal diameter at end-systole (LVIDs) and left ventricular (LV) mass using the software Vevo 2100 (version 1.1.1, Visualsonics, Canada).

### Histological analysis

Masson staining was performed to evaluate collagen volume according to the manufacturer’s instructions (Servicebio Technology, China). The cross-sectional areas of the heart tissues were assessed by wheat germ agglutinin (WGA) staining (Servicebio Technology, China). Cross sections were photographed using a microscope (Leica, Germany), and the data were analyzed with ImageJ software (NIH, United States).

### Evaluation of oxidative stress

Dihydroethidium (DHE) staining solution (Sigma, United States) was added to the frozen sections of heart after dilution in PBS and incubated at 37°C in the dark for 30 min, then the cryosections were rinsed twice with cold PBS. Myocardial tissue homogenate of mice was used to measure malondialdehyde (MDA) and superoxide dismutase (SOD) activity by commercially available MDA and SOD assays (NanJing JianCheng Bioengineering Institute, China). Intracellular MDA activity was measured at 532 nm using a microtiter plate reader (Dragon Wellscan MK2, Finland) and was expressed as nmol/mg of protein. Intracellular SOD activity was measured at 550 nm and was expressed as units/mg of protein.

### Isolation of cardiomyocytes and cardiac fibroblasts

Neonatal rat CMs and CFs were isolated from 0-3-day-old Sprague Dawley rats. In brief, the left ventricles were harvested, minced, and digested with collagenase type II (Worthington Biochemical Corporation, United States) and pancreatin (Sigma, United States) after anesthesia. To remove any contaminating fibroblasts, collected cells were seeded in uncoated 100 mm plastic dishes for 1 h at 37°C in 5% CO_2_ humidified atmosphere. The supernatant, which consists mainly of CMs, was collected and cells were counted and plated on gelatinized 6 well plates 1×10^6^ cells per well. The medium consisted of DMEM/M199 medium (ThermoFisher Scientific, United States) supplemented with 10% FBS, 1% penicillin/streptomycin and 100 μM bromodeoxyuridine. This procedure yields >90% pure CMs. For CFs culture, the supernatant was centrifuged for 5 min (400 x g), resuspended in DMEM medium (ThermoFisher Scientific, United States) supplemented with 10% FBS and plated in 6 well plates. The cells were starved in media containing 0.5% FBS for 12 h, and stimulated with Angiotensin (Ang) II (1 μM) for 24 h (Xu et al., 2019). In this regard, Lu et al, (2022) studied the pharmacokinetic and bioavailability of PRO20 and determined a preferred dosage of 10 nM *in vitro*. Then cells were incubated with PRO20 (10 nM), or thapsigargin (ER stressor, TG, 5 μM) for 24 h (Prola et al., 2017).

### Cardiac fibroblast proliferation and migration analysis

Edu Cell Proliferation Kit (ThermoFisher Scientific, United States) was applied to measure CF proliferation according to the manufacturer’s instructions. After 24-h treatment of DMSO or PRO20, CFs were incubated with Edu (20 μM) for 2 h at 37°C. Following fixation with 4% paraformaldehyde, permeabilized incubation with 0.5% Triton X-100 and staining with Click-iT EdU Alexa Fluor 594 and DAPI. Proliferative CFs were imaged using a fluorescent microscope (Leica, Germany).

The transwell chamber (8 μm pore size, Corning, United States) was applied for CF migration. After PRO20 treatments, CFs were digested with 0.25% trypsin (Hyclone, United States) and resuspended with DMEM without FBS. The 0.6 ml of DMEM with 0.5% FBS was added into lower chamber. Then 100 μl of cell suspension solution was added into the upper chambers and incubated at 37°C for 12 h. After removing the medium, 1 × PBS was used to wash the migrated cells on both side of the membranes and fix the cells with 4% glutaraldehyde for 20 min. CFs that had migrated through the membrane were stained with crystal violet for 30 min and counted with microscope (Leica, Germany).

### RNA isolation and real-time quantitative reverse transcription

Total RNA was extracted from murine left ventricular tissue by TRIzol (Invitrogen, United States) according to the manufacturer’s instructions. Total RNA were reverse transcribed to cDNA with iScriptTM cDNA synthesis Kit (Vazyme, China). Gene expression was analyzed by quantitative PCR using ABI-7900 Real-Time PCR Detection System. The relative expression level was calculated using the 2^−ΔΔCt^ method. GAPDH was used as an internal control. Primers sequences were listed in Supplementary Table S1.

### Western blots

To determine the protein expression levels, cells were lysed with ice-cold RIPA buffer (Beyotime, United States) supplemented with protease and phosphatase inhibitor cocktail (ThermoFisher Scientific, United States), and the protein concentration was quantified via the bicinchoninic acid method. The protocol of western blots was described in previous study (Yuan et al., 2019). The primary antibodies against ANP (diluted 1:1000, Abcam, United States), BNP (diluted 1:1000, Abcam, United States), PRR (diluted 1:500, Abcam, United States), α-SMA (diluted 1:2000, Abcam,United States), Collagen Іα (diluted 1:1000, Abcam, United States), t-IRE1 (diluted 1:1000, Cell Signaling Technology, United States), p-IRE1 (diluted 1:600, Cell Signaling Technology, United States), t-PERK (diluted 1:1000, Cell Signaling Technology, United States), p-PERK (diluted 1:6000, Cell Signaling Technology, United States), ATF-6 (diluted 1:1000, Abcam, United States) and GAPDH (1:5000, SantaCruz, United States) were used in the study. The images were analyzed using Image-Pro Plus software (Version 7.0).

### Analysis of intracellular cAMP and cGMP content

Quantitative determination of intracellular cyclic adenosine-3′,5′-monophosphate (cAMP) and cyclic guanosine-3′,5′-monophosphate (cGMP) was performed according to the manufacture’s introduction (Elabscience, China). The intracellular cAMP and cGMP contents were assessed by measuring absorbance at 450 nm using a microtiter plate reader (Dragon Wellscan MK2, Finland) and calculating from a standard curve.

### Evaluation of soluble (Pro)renin receptor

The plasma content of soluble PRR in mice was determined by enzyme immunoassay kits according to the manufacturer’s instructions (JP27782, IBL, Japan).

### Quantification of angiotensin peptide in the plasma and heart tissues

Plasma was collected in tubes containing PMSF and EDTA. The isolated LV was dissected and homogenized on ice in 0.9% saline/0.1 M HCl containing 0.1 M aprotinin. Total protein content of the homogenate was determined. Then, angiotensin peptide quantification (including Ang I and Ang II) by liquid chromatography tandem-mass spectrometry analysis (LC-MS/MS) was performed in the plasma and heart tissues by Attoquant Diagnostics GmbH (Vienna, Austria) as previously described (Domenig et al., 2016).

### Statistical analysis

Continuous variables were presented as the mean ± standard deviation (SD) for at least three independent experiments. Categorical variables were presented as numbers and percentages. Results were analyzed using GraphPad (SPSS, United States). The significant differences between categorical variables were determined using Chi-square test. The Student’s t test was performed to investigate the difference in continuous variables of normal distribution between two groups. One-way ANOVA was used to compare multiple groups, if appropriate, with Bonferroni correction for post hoc analysis. A *p*-value < 0.05 was considered statistically significant.

## Results

### (Pro)renin receptor expression was upregulated in mouse failing hearts and ang II-induced cardiomyocytes and cardiac fibroblasts

**FIGURE 1:**
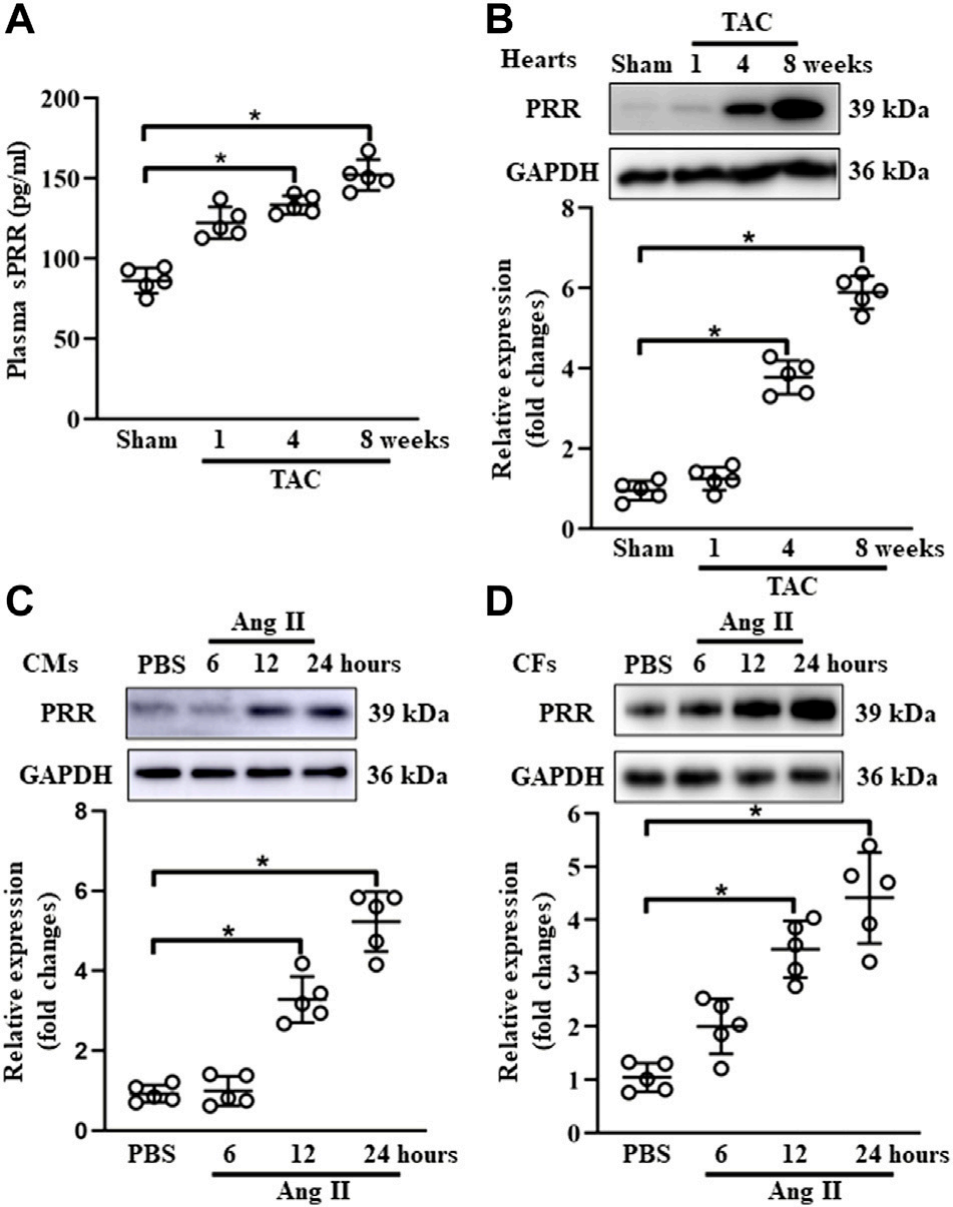
PRR expression is positively associated with cardiac remodeling. **(A)** The plasma concentration of soluble PRR in mice after 1, 4 and 8 weeks of transversus aortic constriction (TAC). **(B)** Immunoblot analysis of PRR in mouse failing hearts and quantitative analysis of the PRR protein level normalized to GAPDH. **(C)** Immunoblot analysis of PRR in Ang II-induced cardiomyocytes (CMs) and quantitative analysis of the PRR protein level normalized to GAPDH. **(D)** Immunoblot analysis of PRR in Ang II-induced cardiac fibroblasts (CFs) and quantitative analysis of the PRR protein level normalized to GAPDH. ^∗^ *p*-value < 0.05. *n* = 5 per group. Data are expressed as mean ± SD.

To explore the potential involvement of PRR in cardiac remodeling, we set up a mouse model of chronic pressure overload-induced cardiac remodeling induced by TAC. As an initial step, we confirmed that heart weight/body weight (HW/BW) was gradually increased at 1-, 4- and 8-weeks post TAC surgery (Supplementary Figure S1A). The expression of hypertrophy-related markers (ANP and BNP) and collagen Iα was significantly upregulated at 4- and 8-weeks after TAC surgery (Supplementary Figure S1B-D). Echocardiography exhibited a reduction of LVEF and FS at 4- and 8-weeks after TAC surgery, while the parameters of cardiac hypertrophy documented as LV mass and LVIDs were increased at 8-weeks post TAC compared with sham group (Supplementary Figure S1E-H). The plasma concentration of soluble PRR was gradually increased in blood from TAC-induced mice (Figure 1A). In comparison with sham group, the protein expression of PRR in hearts was substantially upregulated at 4 and 8 weeks after TAC surgery in mice (Figure 1B). Given the key role of PRR in the formation of angiotensin peptide (Prieto et al., 2021), we also determined the Ang I and II expression in the plasma and heart tissues after TAC surgery. As expected, we found elevated Ang I and II in both plasma and heart tissue lysates in TAC models (Supplementary Figure S2), supporting *in vitro* Ang II induced experiments. Considering the important role of CMs and CFs in TAC-induced hypertrophic mouse hearts, neonatal rat CMs and CFs were isolated and stimulated with Ang II. As shown in Figures 1C,D, the protein expression of PRR in neonatal rat CMs and CFs were gradually increased after AngII treatment for 12 and 24 h. Together, these results indicated that PRR expression was upregulated in both CMs and CFs during TAC-induced cardiac remodeling.

### PRO20 retarded the transverse aortic constriction-induced cardiac remodeling

**FIGURE 2:**
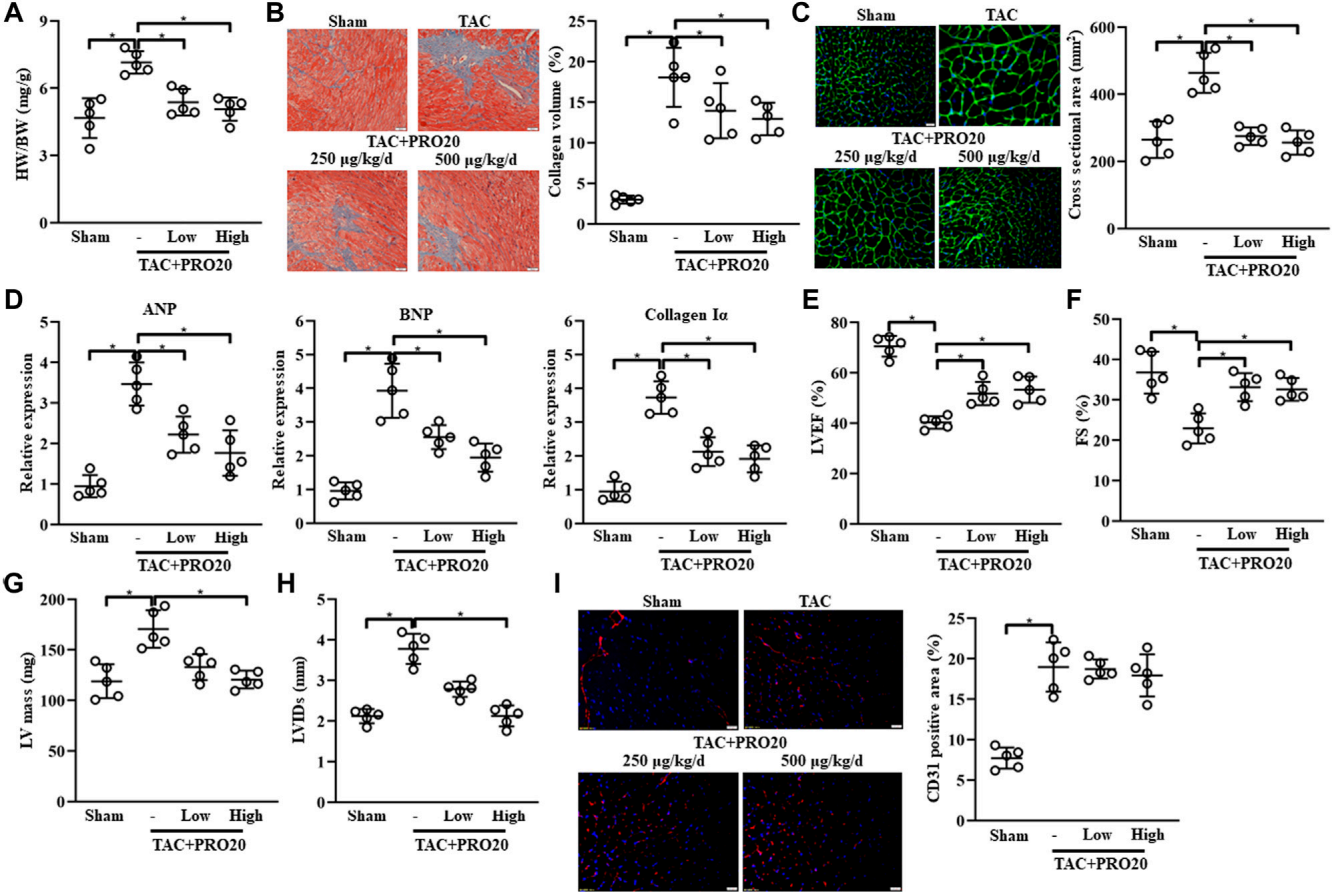
PRR decoy inhibitor PRO20 ameliorates HF in mice after 8 weeks of TAC surgery. **(A)** The ratios of heart weight and body weight (HW/BW) in TAC-induced mice with and without PRO20 treatment for 8 weeks (250 μg/kg/d and 500 μg/kg/d). **(B)** Representative images of Masson staining of heart sections from TAC-induced mice and quantitative analysis of cardiac fibrosis. **(C)** Representative images of wheat germ-agglutinin (WGA) staining of heart sections from TAC-induced mice and quantitative analysis of cardiac hypertrophy. **(D)** Relative mRNA expression of cardiac hypertrophy markers (ANP and BNP) and Collagen Iα in TAC-induced hearts with and without PRO20 treatment. **(E–H)** Echocardiographic analyses of left ventricular ejection fraction (LVEF), fractional shorting (FS), left ventricular mass (LV mass) and left ventricular internal diameter at end-systole (LVIDs) after 8 weeks of TAC operation. **(I)** Representative images of CD31 staining of heart sections from TAC-induced mice and quantitative analysis of angiogenesis. ^∗^ *p*-value < 0.05. n = 5 per group. Data are expressed as mean ± SD.

To investigate the role of PRR in TAC-induced cardiac remodeling, PRR decoy inhibitor PRO20 was synthesized and used to evaluate its inhibitory effect on cardiac remodeling. *In vivo* experiments, PRO20 treatment (250 μg/kg/d or 500 μg/kg/d) successfully attenuated the TAC-induced cardiac remodeling, as indicated by increased HW/BW (Figure 2A) and collagen deposition (Figure 2B). We also confirmed that administration of PRO20 (250 μg/kg/d or 500 μg/kg/d) 1 day after TAC surgery also alleviated cardiac hypertrophy and fibrosis (Supplementary Figure S3A, S3B). Furthermore, we measured the cross-sectional area (CSA) using WGA staining and found that PRO20 treatment retarded TAC-induced cardiac hypertrophy (Figure 2C). Moreover, increased mRNA expression of cardiac hypertrophic markers (ANP, BNP) and fibrotic marker (collagen Iα) induced by TAC were significantly reduced after PRO20 treatment (Figure 2D). In addition, PRO20 administration improved TAC-mediated LV dilation and dysfunction as evidenced by results of LVEF, FS, LV mass and LVIDs (Figures 2E–H). However, results of CD31 staining showed that PRO20 treatment did not affect angiogenesis in hearts of TAC mice (Figure 2I). Taken together, these results indicated that PRR decoy inhibitor PRO20 (250 μg/kg/d or 500 μg/kg/d) retarded the TAC-induced cardiac hypertrophy and fibrosis.

### PRO20 suppressed oxidative stress in transverse aortic constriction-induced hypertrophic hearts

**FIGURE 3:**
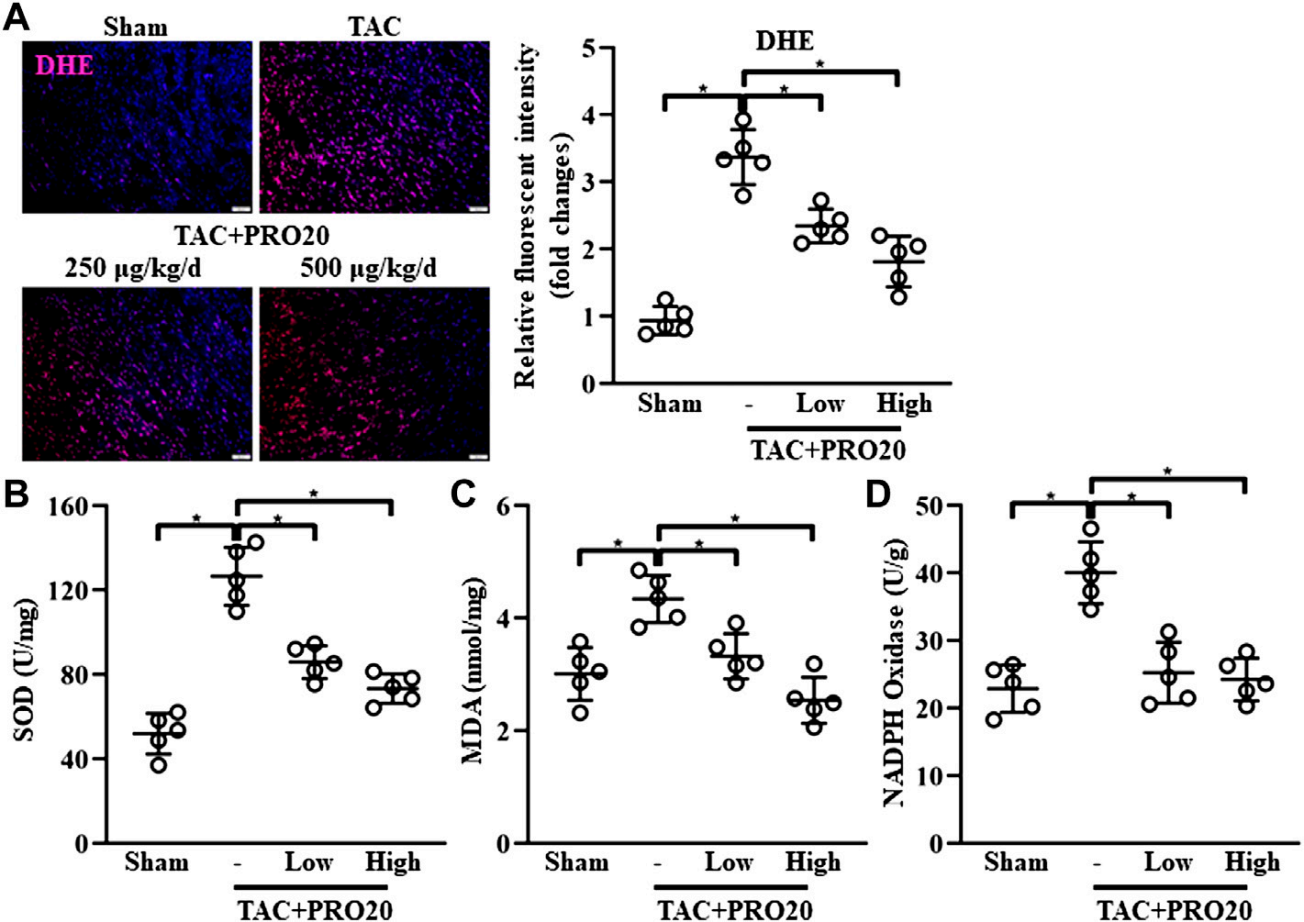
PRR decoy inhibitor PRO20 suppresses oxidative stress in TAC-induced mice. **(A)** Representative immunofluorescent images of the production of reactive oxygen species assessed by dihydroethidium (DHE, stained in red) in hearts from TAC-induced mice with and without PRO20 treatment for 8 weeks. Nuclei were counterstained with DAPI (blue). **(B–D)** The content of SOD, MDA and NADPH oxidase in hearts from TAC-induced mice with and without PRO20 treatment for 8 weeks ^∗^ *p*-value < 0.05. n = 5 per group. Data are expressed as mean ± SD.

Considering the detrimental effect of PRR on oxidative stress response (Dong et al., 2019), we examined whether PRO20 could inhibit oxidative stress in TAC-induced hypertrophic hearts. DHE staining was used to assess ROS levels, indicating that the intracellular content of ROS production was substantially elevated from 1-week post TAC surgery (Supplementary Figure S4A). Results showed that PRO20 significantly suppressed the ROS generation in hypertrophic hearts induced by TAC (Figure 3A). Further, superoxide dismutase (SOD) activity, malondialdehyde (MDA) content and NADPH oxidase activity were measured after PRO20 treatment. As expected, PRO20 treatment significantly reduced the elevated SOD activity, MDA content and NADPH oxidase activity in hypertrophic hearts (Figures 3B–D). These findings indicated that PRO20 suppressed TAC-induced oxidative stress response.

### PRO20 inhibited cardiomyocyte hypertrophy and alleviated fibroblast activation

**FIGURE 4:**
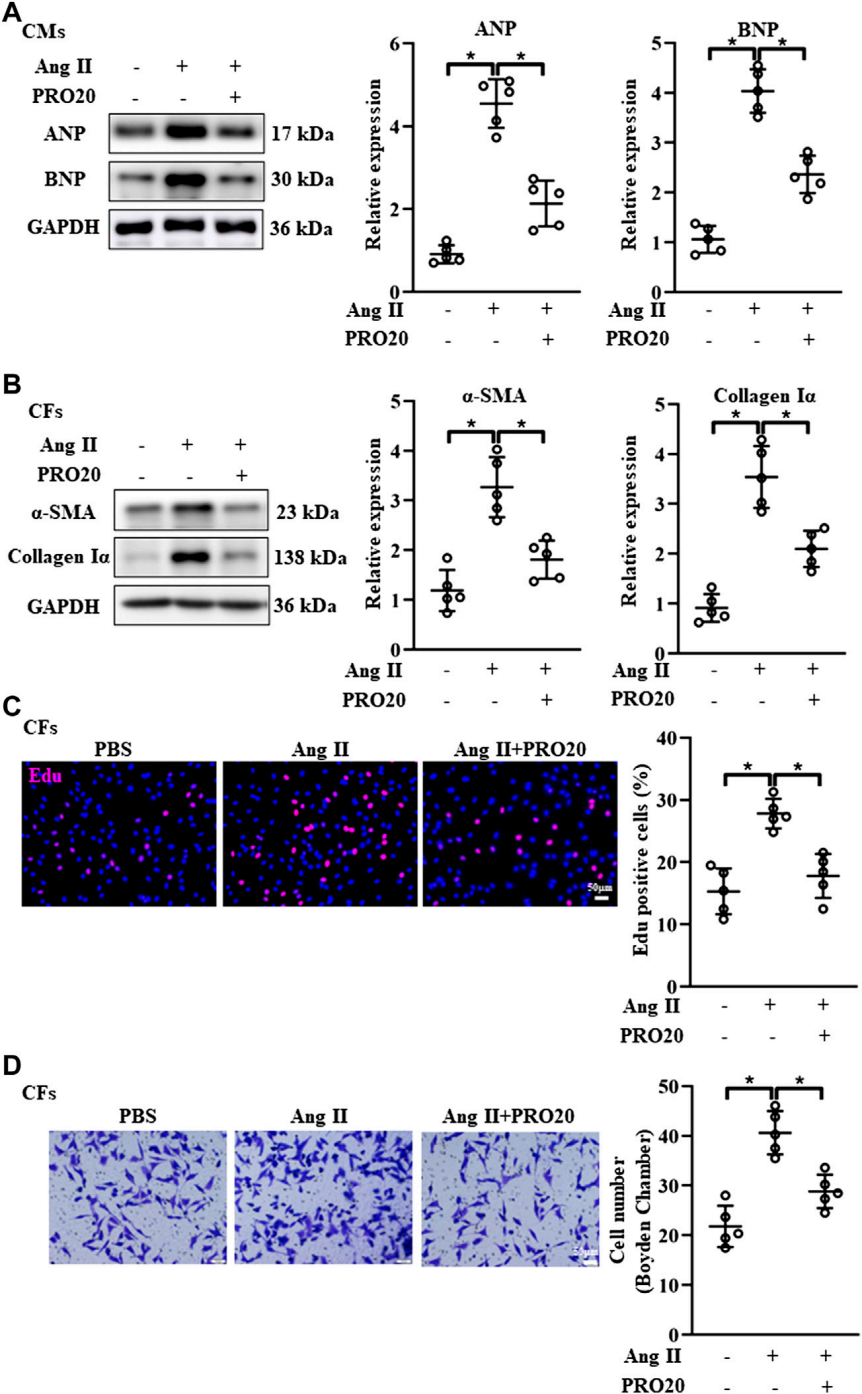
Inhibition of PRR by PRO20 alleviates CM hypertrophy and CF activation. **(A)** Immunoblot analysis of ANP and BNP in Ang II-induced CMs and quantitative analysis of the ANP and BNP protein level normalized to GAPDH. **(B)** Immunoblot analysis of α-SMA and Collagen Iα in Ang II-induced CFs and quantitative analysis of the α-SMA and Collagen Iα protein level normalized to GAPDH. **(C)** Effect of PRO20 on CF proliferation assessed by Edu assay. **(D)** Boyden chamber assay was performed to evaluate the migration of CFs with and without PRO20 treatment (10 nM) in the presence of Ang II. ^∗^ *p*-value < 0.05. n = 5 per group. Data are expressed as mean ± SD.

It is well established that cardiomyocyte hypertrophy and fibroblast activation play important roles in pathological cardiac remodeling (Chen et al., 2020; Vigil-Garcia et al., 2021). Then, we isolated the CMs and CFs *in vitro*, and evaluate the influence of PRO20 on these cells. In line with the findings *in vivo*, PRO20 treatment inhibited Ang II-induced cardiomyocyte hypertrophy, as indicated by the protein expression of ANP and BNP (Figure 4A). With regard to fibroblast activation, we first examined the expression of fibrotic markers, and results showed that Ang II increased the αSMA and collagen Iα, which was successfully attenuated by PRO20 administration (Figure 4B). Then, *in vitro* cell proliferation and migration of CFs were determined using Edu staining and Transwell assay. We found that PRO20 significantly suppressed Ang II-mediated cell proliferation and migration in CFs (Figures 4C,D). These findings indicated that PRO20 attenuated pathological cardiac remodeling through inhibition of cardiomyocyte hypertrophy and fibroblast activation.

### PRO20 enhanced intracellular cAMP levels and attenuated endoplasmic reticulum stress

**FIGURE 5:**
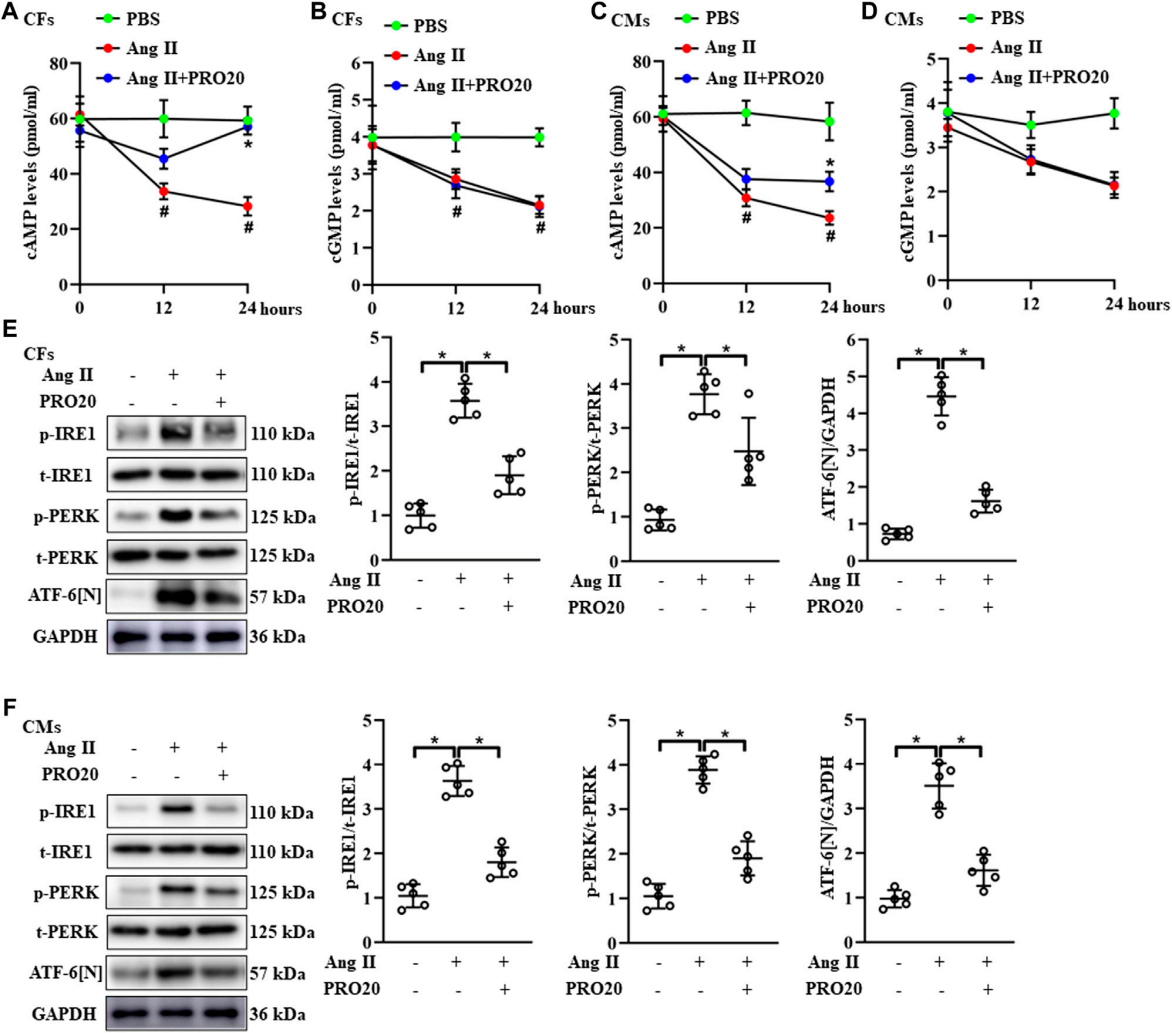
PRR decoy inhibitor PRO20 promotes intracellular cAMP content and suppresses endoplasmic reticulum stress. **(A–B)** The intracellular content of cAMP and cGMP in Ang II-induced CFs with and without PRO20 treatment (10 nM). #*p* < 0.05 vs. PBS group; ^∗^ *p*-value < 0.05 vs. Ang II group at indicated time point. **(C–D)** The intracellular content of cAMP and cGMP in Ang II-induced CMs with and without PRO20 treatment (10 nM). #*p* < 0.05 vs. PBS group; ^∗^ *p*-value < 0.05 vs. Ang II group at indicated time point. **(E)** Immunoblot analysis of IRE1, PERK and cleaved N-terminal cytoplasmic domain of ATF-6 (ATF-6 [N]) in Ang II-induced CFs and quantitative analysis of the IRE1 and PERK phosphorylation and the ATF-6 [N] activation. ^∗^ *p*-value < 0.05. **(F)** Immunoblot analysis of IRE1, PERK and ATF-6 [N] in Ang II-induced CMs and quantitative analysis of the IRE1 and PERK phosphorylation and the ATF-6 [N] activation. ^∗^ *p*-value < 0.05. n = 5 per group. Data are expressed as mean ± SD.

Since the cyclic nucleotides cAMP and cGMP were well-characterized second messenger molecules regulating many intracellular processes, such as cell proliferation, migration (Wolter et al., 2017), we further explored whether PRO20 inhibited pathological cardiac remodeling through modulating intracellular cAMP and cGMP levels. Upon Ang II stimulation in CFs and CMs, intracellular cAMP and cGMP levels were significantly decreased (Figures 5A–D). Of note, PRO20 treatment only reversed the upregulated cAMP levels induced by Ang II treatment in CFs and CMs, but did not affect intracellular cGMP levels (Figures 5A–D). These results suggested that PRO20 enhanced intracellular cAMP levels in CFs and CMs, but not cGMP levels.

**FIGURE 6:**
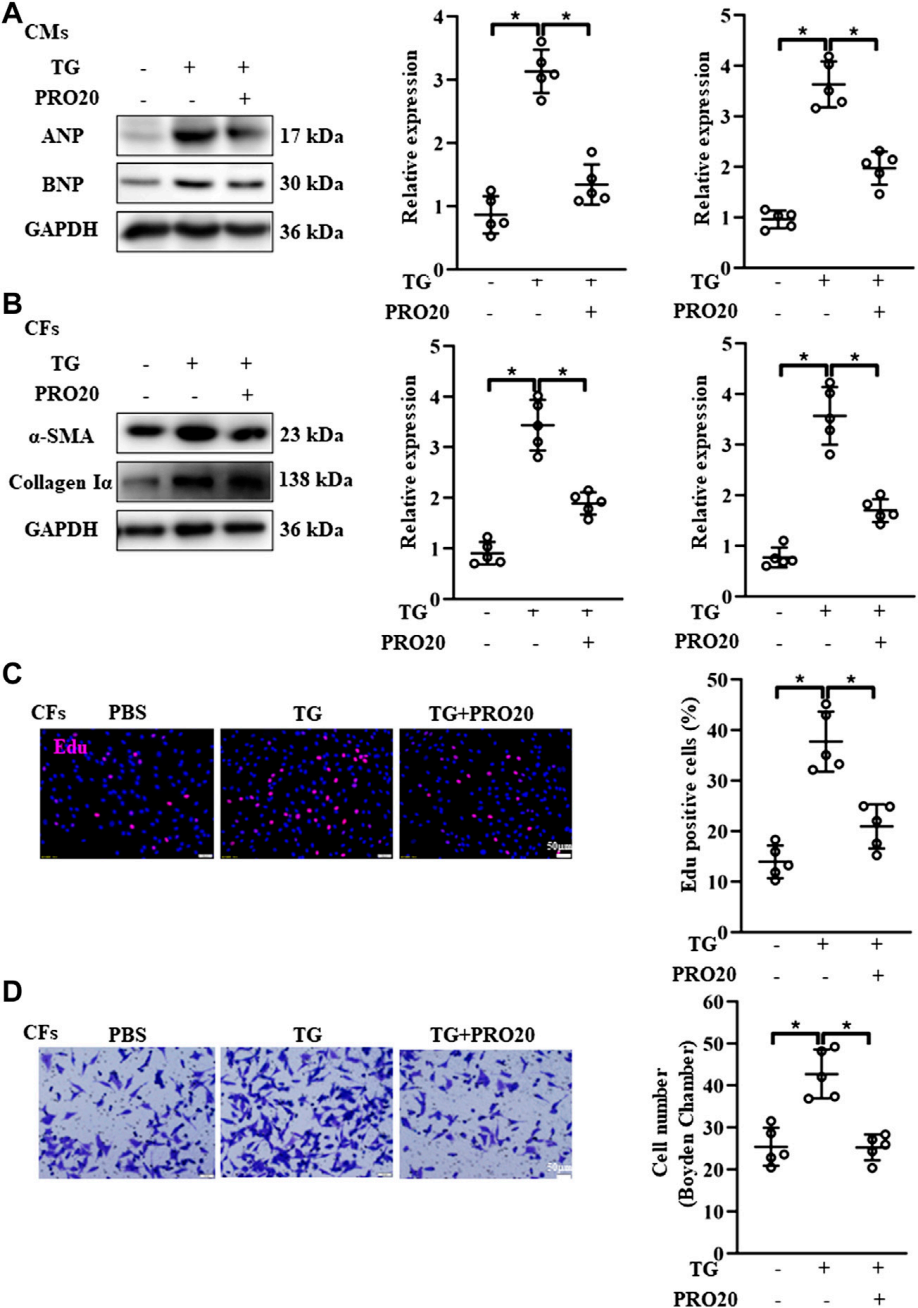
Effects of PRO20 on CM hypertrophy and CF activation induced by ER stressor TG. **(A)** Immunoblot analysis of ANP and BNP in TG-induced (5 μM) CMs with and without PRO20 treatment and quantitative analysis of the ANP and BNP protein level normalized to GAPDH. **(B)** Immunoblot analysis of α-SMA and Collagen Iα in CFs with and without TG treatment and quantitative analysis of the α-SMA and Collagen Iα protein level normalized to GAPDH. **(C)** Effect of PRO20 on TG-induced CF proliferation assessed by Edu assay in indicated groups. **(D)** Boyden chamber assay was performed to evaluate the migration of CFs in indicated groups. ^∗^ *p*-value < 0.05. n = 5 per group. Data are expressed as mean ± SD.

Accumulating evidence showed that RAS activation promotes ER-stress related disease (Wu et al., 2017; Yang et al., 2017). Likewise, the phosphorylation of IRE1 and PERK, as well as ATF-6 [N] expression, were upregulated in hearts after TAC operation (Supplementary Figure S4B). Next, we investigated the effects of PRO20 on ER stress, including phosphorylation of IRE1 and PERK and ATF-6 cleavage. In CFs and CMs, Ang II administration significantly upregulated the activation of ATF-6 [N] and the phosphorylation of IRE1 and PERK as well, which was successfully abolished by PRO20 treatment (Figures 5E,F). To further verify whether PRO20 inhibits cardiac remodeling through ER stress, CMs and CFs were incubated with ER stressor TG in the presence of PRO20. Indeed, PRO20 was able to reverse TG-induced CM hypertrophy, CF activation and migration (Figures 6A,B). The findings indicated that PRO20 suppressed Ang II-induced ER stress in CFs and CMs.

## Discussion

**FIGURE 7:**
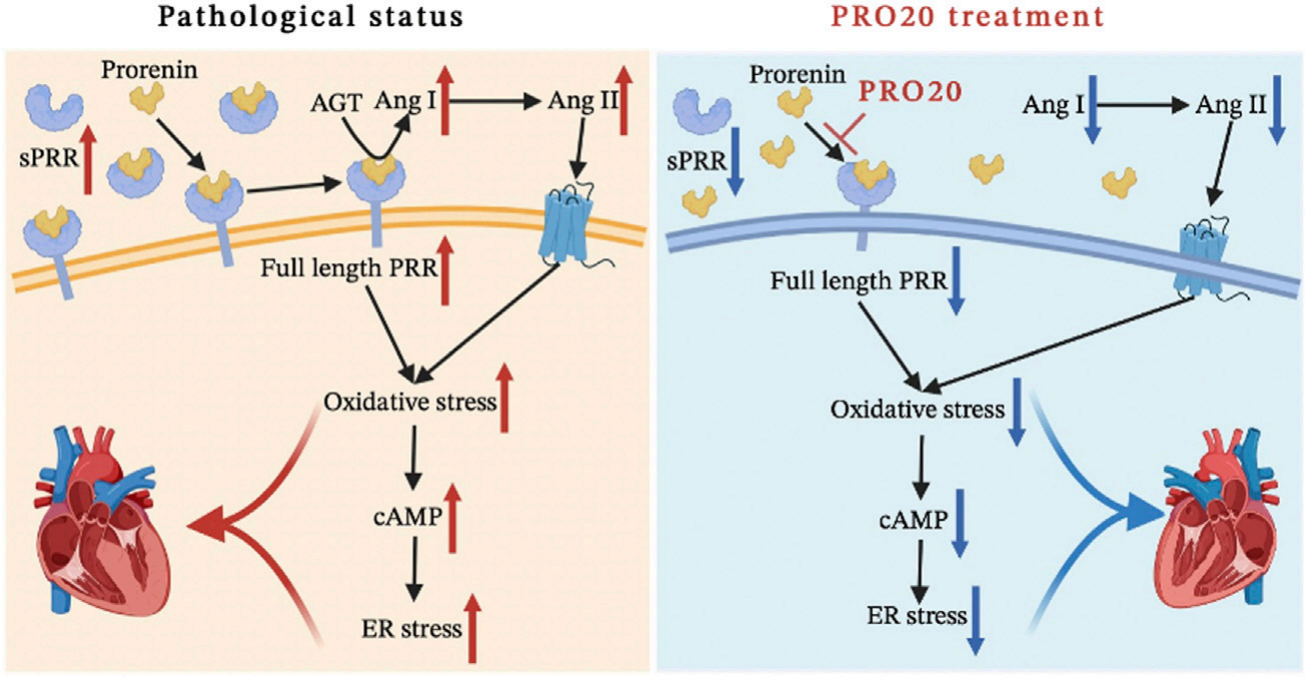
Schematic diagram of the mechanism. We systematically analyzed the mechanism of the PRO20 effect on cardiac remodeling, and found that PRO20 inhibited both oxidative stress and ER stress signaling pathways.

In the present study, we demonstrated for the first time that the importance of PRR during pathological cardiac remodeling in response to chronic pressure overload. We showed that PRR decoy inhibitor PRO20 ameliorated cardiomyocyte hypertrophy and fibroblast activation through suppression of oxidative stress, enhancement of intracellular cAMP levels and inhibition of ER stress *in vivo* and *in vitro* (Figure 7). These findings suggested that PRR decoy inhibitor PRO20 might serve as a potent pharmaceutical for pathological cardiac remodeling.

HF as a burgeoning problem affect more than 20 million individuals worldwide (Wu et al., 2017). However, mechanisms contributing to the development of pathological cardiac remodeling remains incompletely understood. Hence, HF remains challenging to treat, and there is an urgent need to identify new therapeutics. During the pathological cardiac remodeling process, cardiomyocytes integrate several signals from extracellular matrix, cell membrane, ion channels, cytoskeleton, sarcomere, mitochondria and ER to translate biomechanical forces to fetal gene expression, which contributes to alteration in cardiomyocyte shape and structure (Haque and Wang, 2017). ER as a multifunctional intracellular organelle, plays an important role in protein synthesis, protein folding and translocation. Pathological processes that disturb protein folding in ER lumens caused ER stress (Doyle et al., 2011). In response to ER stress, unfolded protein response is activated by three major ER membrane associated proteins, including IRE1, PERK, ATF6 (Zhang et al., 2017). Once activated, these sensors stimulate downstream signaling pathways to inhibit mRNA translation and protein load on the ER and facilitate retrotranslocation of proteins from the ER to the cytosol for proteasomal degradation. Notably, ATF-6 and IRE1 activation can directly upregulated the unfolded protein response gene mRNA transcription or processing (Lee et al., 2002; Yamamoto et al., 2007), while PERK inhibits the initiation and decreases the protein load on the ER (Lee et al., 2002). ATF-6, IRE1 and PERK-induced deterioration of the Ca^2+^ ion balance leads to cardiac hypertrophy caused by hyperthyroidism (Bektur Aykanat et al., 2021), and PERK was also essential during the experiments for avoiding TAC-induced congestive HF (Liu et al., 2014). In line with these findings, we found that there was a remarkable change in the activation of IRE1, PERK and ATF-6 in Ang II-treated CMs and CFs, suggesting that the ER stress induced by Ang II participated in cardiomyocyte hypertrophy and fibroblast activation.

PRR as a member of RAS, activated prorenin and enhance the enzymatic activity of renin, and then promotes Ang II formation (Hennrikus et al., 2018). Numerous studies showed that PRR activation could stimulate intracellular pathways related to the cardiac damage (Mahmud et al., 2012; Hennrikus et al., 2018). In the heart, PRR not only promotes atrial structure and electrical remodeling (Lu et al., 2016), but also augments the cardiac damage in dilated cardiomyopathy (Mahmud et al., 2012) and diabetic cardiomyopathy (Dong et al., 2019). Further, a recent clinical study also showed that serum soluble PRR concentrations was associated with the severity of HF (Gong et al., 2019). Consistently, we observed significant increase of PRR protein levels and elevated plasma concentration of soluble PRR after TAC surgery. Feng et al. (Feng et al., 2021) revealed that Ang II activated PRR in inner medulla but did not affect their expression in renal cortex. In line with these findings, our results indicated that Ang II was also capable of stimulating PRR in hearts, implying a positive feedback between PRR and Ang II under pathological conditions such as hypertension and heart failure. Further studies are required to investigate their underlying mechanisms and distinguish whether the positive feedback was an autonomous or systematic manner. All these findings indicated that the PRR downregulation might be a therapeutic target for pathological cardiac remodeling.

Accumulating evidence indicates that inhibition of PRR ameliorates the cardiac fibrosis and impairment in cardiac function via reduction of ROS generation (Ellmers et al., 2016; Dong et al., 2019). PRR decoy inhibitor PRO20 (the first 20 amino acid residues of the prorenin prosegment) is a specific PRR ligand that blocks activation of prorenin by binding to the PRR (Li et al., 2015a). PRO20 not only attenuates prorenin, but also retards the development of DOCA-salt-induced hypertension and Ang II-dependent hypertension (Li et al., 2015a). However, the effects of PRO20 on pressure overload-induced pathological cardiac hypertrophy remain unknown. Therefore, in current study, TAC surgery was conducted to induce cardiac remodeling, followed by systematic administration of PRO20. As expected, PRO20 administration attenuated the pressure overload-induced oxidative stress during pathological cardiac remodeling. Further, PRO20 treatment in CMs and CFs produced similar RAS-suppressing effects, including suppression of cardiomyocyte hypertrophy and attenuation of fibroblast activation.

The second messengers cAMP and cGMP serve as the key regulators of cardiac remodeling, and mediate heart failure pathophysiology induced by different forms of injury and stress. As siblings, cardiac cAMP synthesis is catalyzed by adenylyl cyclases principally in response to adrenergic receptor stimulation, while production of cGMP is initiated by guanylyl cyclases, which are sensitive to either nitric oxide or natriuretic peptides (Preedy, 2020). Commonly, cAMP regulates the strength and frequency of cardiac contraction and relaxation, whereas cGMP modulates inotropy and metabolic responses (Chen and Yan, 2021). Further, the cyclic nucleotide phosphodiesterases (PDEs) facilitate cyclic nucleotide degradation of cAMP and cGMP, and are vital for mediating crosstalk between the two pathways. Several studies reported that Ang II mediates cAMP decrease (Li et al., 2006; Crajoinas et al., 2016), but some studies also observed that Ang II treatment has no effect on cAMP levels (Cano et al., 1994). These differences might be attributable to different cells in response to various stimuli. With regard to cGMP, augmentation of cGMP signaling is recommended as a potential therapeutic strategy in HF based on several preclinical and clinical studies that have investigated various mechanisms and effects of cGMP enhancement (Greene et al., 2013; Richards et al., 2021; Tripathi et al., 2021). In our present study, Ang II treatment significantly reduced intracellular cAMP and cGMP levels in both CMs and CFs. Ang II upregulates PRR expression both in cultured cells and in DOCA-salt hypertensive mice through activation of cAMP response element-binding protein (Li et al., 2015b). Besides, Huang et al. found that sodium depletion upregulates renal PRR expression via cGMP-protein kinase G signaling pathway (Huang and Siragy, 2012). Nevertheless, whether PRR inhibition by PRO20 could affect the intracellular cAMP and cGMP levels is still unknown. Through a series of *in vitro* experiments, we found that PRO20 only reversed Ang II-induced upregulation of cAMP levels in CMs and CFs, but did not affect the Ang II-mediated downregulation of cGMP levels. As to these discrepancies, the main reason responsible for them may be that PRO20 treatment affects cAMP-specific synthesis and/or degradation, but not cGMP. Further investigation is required to clarify whether PRO20 enhances cAMP through interplaying with specific PDEs or adenylyl cyclases.

In summary, PRR as an important component of RAS, is enrolled in pathological cardiac remodeling, and PRR decoy inhibitor PRO20 attenuated pressure overload induced cardiomyocyte hypertrophy and fibroblast activation through inhibition of the ROS generation and ER stress in CMs and CFs.

## Data Availability

The original contributions presented in the study are included in the article/Supplementary Materials, further inquiries can be directed to the corresponding author.
