# Supplementary material for: Inhibitory effect of (pro)renin receptor decoy inhibitor PRO20 on endoplasmic reticulum stress during cardiac remodeling
Source: Front Pharmacol. 2022 Aug 12;13:940365. doi: 10.3389/fphar.2022.940365 (PMC9411812; doi:10.3389/fphar.2022.940365)
Supplement: Supplementary file 1 [file Table1.DOCX]

| Gene | Primers (5’-3’) |
| --- | --- |
| ANP | Forward: 5’-GCT TCC AGG CCA TAT TGG AG-3’  Reverse: 5’-GGG GGC ATG ACC TCA TCT T-3’ |
| BNP | Forward: 5’-GAG GTC ACT CCT ATC CTC TGG-3’  Reverse: 5’-GCC ATT TCC TCC GAC TTT TCT C-3’ |
| Collagen Ia | Forward: 5’-GCT CCT CTT AGG GGC CAC T-3’  Reverse: 5’-CCA CGT CTC ACC ATT GGG G-3’ |
| GAPDH | Forward: 5’-AGG TCG GTG TGA ACG GAT TTG-3’  Reverse: 5’-TGT AGA CCA TGT AGT TGA GGT CA-3’ |

Table S1. Primer sequences for real-time quantitative PCR.
