# Supplementary material for: Inhibitory effect of (pro)renin receptor decoy inhibitor PRO20 on endoplasmic reticulum stress during cardiac remodeling
Source: Front Pharmacol. 2022 Aug 12;13:940365. doi: 10.3389/fphar.2022.940365 (PMC9411812; doi:10.3389/fphar.2022.940365)
Supplement: Supplementary file 2 [file DataSheet2.docx]

**Inhibitory effect of (pro)renin receptor decoy inhibitor PRO20 on endoplasmic reticulum stress during cardiac remodeling**

***Running title: PRO20 on cardiac remodeling***

**Authors:** Jing Zhang^1^, Yun-Jiu Cheng^2^, Chang-Jun Luo^1^, Jia Yu^3, *^

1 Department of Cardiology, Liuzhou Municipal Liutie Central Hospital, Guangxi, China

2 Department of Cardiology, The First Affiliated Hospital of Sun Yat-sen University, Guangdong, China

3 Department of General Practice School, Guangxi Medical University, Guangxi, China

* Correspondence: Jia Yu, MD, Department of General Practice School, Guangxi Medical University, Guangxi, China. Email: [yujia@gxmu.edu.cn](mailto:yujia@gxmu.edu.cn)

The raw data have been uploaded on Nutstore. The links are presented as follows.

Stainings: <https://www.jianguoyun.com/p/DaF941kQtKOvChjZnbMEIAA>

Original gels: <https://www.jianguoyun.com/p/DXU4FFUQtKOvChjfnbMEIAA>

Script files for GraphPad:

<https://www.jianguoyun.com/p/DYCmSD0QtKOvChjnnbMEIAA>
