# Supplementary material for: Inhibitory effect of (pro)renin receptor decoy inhibitor PRO20 on endoplasmic reticulum stress during cardiac remodeling
Source: Front Pharmacol. 2022 Aug 12;13:940365. doi: 10.3389/fphar.2022.940365 (PMC9411812; doi:10.3389/fphar.2022.940365)
Supplement: Supplementary file 3 [file DataSheet1.docx]

**Supplementary figures and figure legends:**

**Supplementary Figure 1**


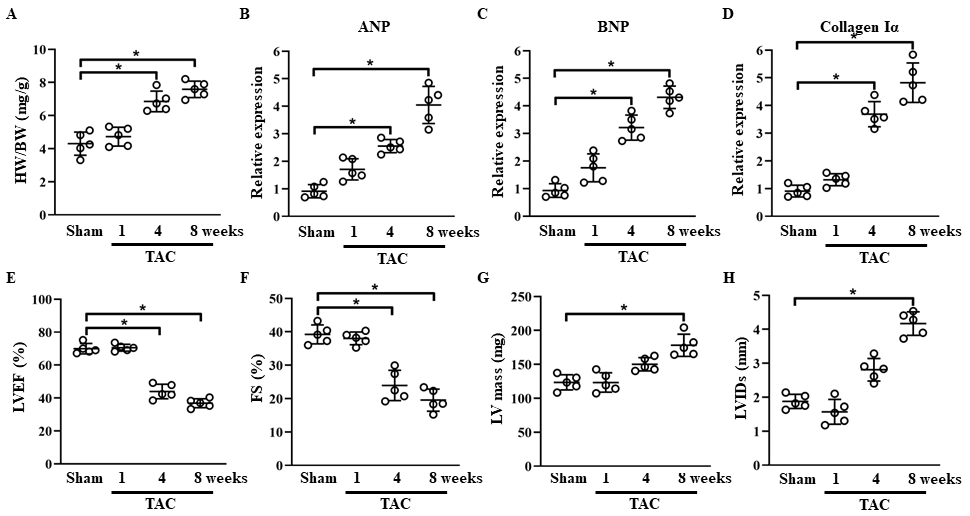


**Supplementary Figure 1. TAC surgery gradually induces HF in mice.**

(A) The ratios of heart weight and body weight (HW/BW) at 1-, 4- and 8-week post TAC surgery. (B-D) Relative mRNA expression of cardiac hypertrophy markers (ANP and BNP) and Collagen Iα in the hearts at 1-, 4- and 8-week post TAC surgery. (E-H) Echocardiographic analyses of left ventricular ejection fraction (LVEF), fractional shorting (FS), left ventricular mass (LV mass) and left ventricular internal diameter at end-systole (LVIDs) at 1-, 4- and 8-week post TAC surgery. n = 5 per group. Data are expressed as mean ± SD. * P-value < 0.05.

**Supplementary Figure 2**

**
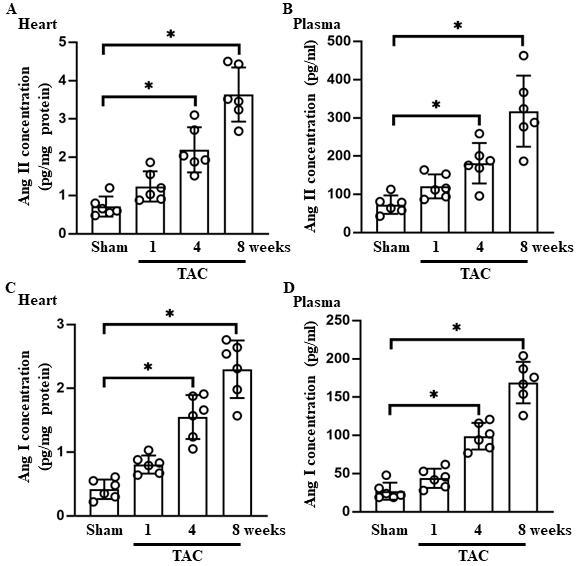
**

**Supplementary Figure 2. Angiotensin peptide levels in the plasma and heart tissues after TAC surgery.**

(A-D) Ang I and II expression in the plasma and heart tissues at 1-, 4- and 8-week post TAC surgery. n = 6 per group. Data are expressed as mean ± SD. * P-value < 0.05.

**Supplementary Figure 3.**

**
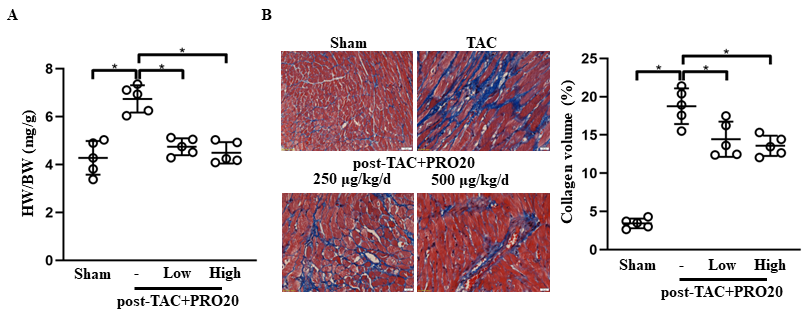
**

**Supplementary Figure 3. Post-TAC administration of PRR decoy inhibitor PRO20 alleviates cardiac remodeling.**

(A) The ratios of heart weight and body weight (HW/BW) after post-TAC administration of PRO20 (250 μg/kg/d and 500 μg/kg/d). (B) Representative images of Masson staining of heart sections after post-TAC administration of PRO20 (250 μg/kg/d and 500 μg/kg/d) and quantitative analysis of cardiac fibrosis. n = 5 per group. Data are expressed as mean ± SD. * P-value < 0.05.

**Supplementary Figure 4**

**
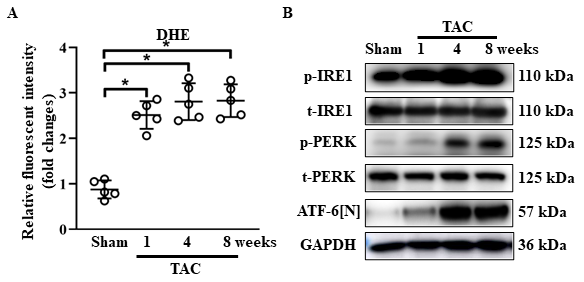
**

**Supplementary Figure 4. TAC surgery induces oxidative stress and ER stress in murine hearts.**

(A) Quantification of DHE staining of murine hearts at 1-, 4- and 8-week post TAC surgery. (B) Immunoblot analysis of IRE1, PERK and cleaved N-terminal cytoplasmic domain of ATF-6 (ATF-6[N]) in Sham-/TAC-induced murine hearts. n = 5 per group. Data are expressed as mean ± SD. * P-value < 0.05.
